# Molecular characterization of CNS paragangliomas identifies cauda equina paragangliomas as a distinct tumor entity

**DOI:** 10.1007/s00401-020-02218-7

**Published:** 2020-09-14

**Authors:** Leonille Schweizer, Felix Thierfelder, Christian Thomas, Patrick Soschinski, Abigail Suwala, Damian Stichel, Annika K. Wefers, Lars Wessels, Martin Misch, Hee-yeong Kim, Ruben Jödicke, Daniel Teichmann, David Kaul, Johannes Kahn, Michael Bockmayr, Martin Hasselblatt, Alexander Younsi, Andreas Unterberg, Bettina Knie, Jan Walter, Diaa Al Safatli, Sven-Axel May, Andreas Jödicke, Georgios Ntoulias, Dag Moskopp, Peter Vajkoczy, Frank L. Heppner, David Capper, Wolfgang Hartmann, Christian Hartmann, Andreas von Deimling, David E. Reuss, Anne Schöler, Arend Koch

**Affiliations:** 1 Department of Neuropathology, Berlin Institute of Health, Charité-Universitätsmedizin Berlin, Corporate Member of Freie Universität Berlin, Humboldt-Universität Zu Berlin, Charitéplatz 1, 10117 Berlin, Germany; 2 German Cancer Consortium (DKTK), Partner Site Berlin, German Cancer Research Center (DKFZ), Heidelberg, Germany; 3 Institute of Neuropathology, University Hospital Münster, Pottkamp 2, Münster, Germany; 4 Department of Neuropathology, Institute of Pathology, Heidelberg University Hospital, Heidelberg, Germany; 5 Clinical Cooperation Unit Neuropathology, German Cancer Research Center (DKFZ), German Consortium for Translational Cancer Research (DKTK), Heidelberg, Germany; 6 Hopp Children’s Cancer Center (KiTZ), Heidelberg, Germany; 7 Department of Neurosurgery, Charité-Universitätsmedizin Berlin, Berlin, Germany; 8 Department of Radiation Oncology and Radiotherapy, Charité Universitätsmedizin Berlin, Berlin, Germany; 9 Department of Radiology, Charité Universitätsmedizin Berlin, Berlin, Germany; 10 Department of Pathology, Berlin Institute of Health, Charité-Universitätsmedizin Berlin, Corporate Member of Freie Universität Berlin, Humboldt-Universität Zu Berlin, Berlin, Germany; 11 Department of Pediatric Hematology and Oncology, University Medical Center Hamburg-Eppendorf, Hamburg, Germany; 12 Research Institute Children’s Cancer Center Hamburg, Hamburg, Germany; 13 Department of Neurosurgery, Heidelberg University Hospital, Heidelberg, Germany; 14 Department of Neurosurgery, Vivantes Klinikum Im Friedrichshain, Berlin, Germany; 15 Department of Neurosurgery, Universitätsklinikum Jena, Jena, Germany; 16 Department of Neurosurgery, Klinikum Chemnitz, Chemnitz, Germany; 17 Department of Neurosurgery, Vivantes Klinikum Neukölln, Berlin, Germany; 18 Division of Translational Pathology, Gerhard-Domagk-Institute of Pathology, University Hospital Münster, Münster, Germany; 19 Department of Neuropathology, Institute of Pathology, Hannover Medical School, Hannover, Germany; 20 Cluster of Excellence, NeuroCure, Charitéplatz 1, 10117 Berlin, Germany; 21 German Center for Neurodegenerative Diseases (DZNE) Berlin, 10117 Berlin, Germany

**Keywords:** Paraganglioma, Cauda equina, Head and neck, SDHB, GATA3, DNA methylation

## Abstract

Paragangliomas/pheochromocytomas are rare neuroendocrine tumors that arise from the adrenal gland or ganglia at various sites throughout the body. They display a remarkable diversity of driver alterations and are associated with germline mutations in up to 40% of the cases. Comprehensive molecular profiling of abdomino-thoracic paragangliomas revealed four molecularly defined and clinically relevant subtypes. Paragangliomas of the cauda equina region are considered to belong to one of the defined molecular subtypes, but a systematic molecular analysis has not yet been performed. In this study, we analyzed genome-wide DNA methylation profiles of 57 cauda equina paragangliomas and show that these tumors are epigenetically distinct from non-spinal paragangliomas and other tumors. In contrast to paragangliomas of other sites, chromosomal imbalances are widely lacking in cauda equina paragangliomas. Furthermore, RNA and DNA exome sequencing revealed that frequent genetic alterations found in non-spinal paragangliomas—including the prognostically relevant SDH mutations—are absent in cauda equina paragangliomas. Histologically, cauda equina paragangliomas show frequently gangliocytic differentiation and strong immunoreactivity to pan-cytokeratin and cytokeratin 18, which is not common in paragangliomas of other sites. None of our cases had a familial paraganglioma syndrome. Tumors rarely recurred (9%) or presented with multiple lesions within the spinal compartment (7%), but did not metastasize outside the CNS. In summary, we show that cauda equina paragangliomas represent a distinct, sporadic tumor entity defined by a unique clinical and morpho-molecular profile.

**Electronic supplementary material:**

The online version of this article (10.1007/s00401-020-02218-7) contains supplementary material, which is available to authorized users.

## Introduction

**Fig. 1:**
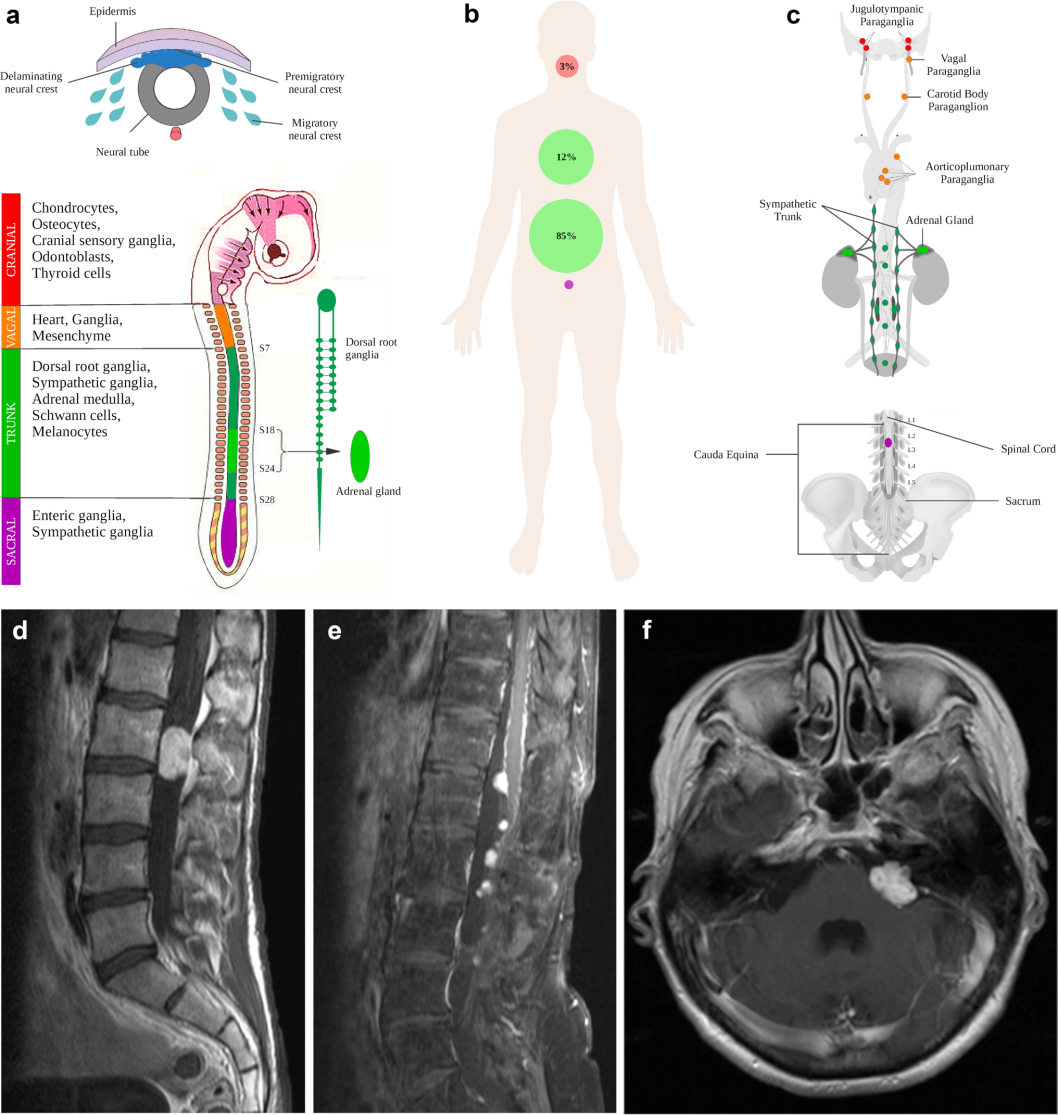
Developmental, clinical and radiological characteristics of paragangliomas. **a** Paraganglia develop from neural crest cell precursor cells that migrate to various body sites. Different subpopulations are recognized that differ in their spatial predominance and capability of giving rise to functional or non-functional paragangliomas. **b** Distribution and frequency of paragangliomas in the human body: cauda equina paragangliomas are exceptionally rare. **c** Common sites of paraganglioma origin in detail with type of precursor neural crest cell population indicated by color. **d** Typical MRI presentation of a paraganglioma in the cauda equina region as a circumscribed, oval-shaped, contrast-enhancing mass at L2 on a sagittal T1 image with contrast agent. **e** Paraganglioma with unusual dissemination up to the thoracic spine upon tumor recurrence (sagittal T1 image with contrast agent). More often, dissemination occurs within cauda equina nerve roots in the lumbar compartment. **f** Same patients as in (**e**) with a paraganglioma metastasis in the cerebellopontine angle—mimicking a glomus jugulare tumor (axial T1 image with contrast agent)

Paragangliomas and pheochromocytomas are rare, neuroendocrine tumors developing from the autonomous nervous system. Arising in the adrenal medulla, tumors are called pheochromocytomas (PCC), whereas tumors in extra-adrenal location are called paragangliomas (PGL) [5]. Paraganglia are derived of different migrating neural crest cell subpopulations (Fig. 1a), giving rise to either sympathetic, catecholamine-secreting PGLs (mainly abdomino-thoracic) or non-secreting, parasympathetic PGL (mainly head and neck PGL).

Paragangliomas of the CNS almost exclusively occur in the cauda equina region or the cerebellopontine angle, often referred to as glomus jugulare tumors or jugulotympanic paragangliomas [13]. Compared to the more prevalent abdominal (85%), thoracic (12%) and head and neck (3%) PGLs, paragangliomas in the cauda equina region (CEP) are exceptionally rare (< 1% of all PGLs, Fig. 1b), accounting for only 3–4% of all tumors arising in the lower lumbar spine [37]. Despite their different anatomic locations, PGLs share characteristic morphological and ultrastructural features and are considered to belong to the same tumor entity [7, 13], although the histogenesis of CEPs is unclear and paraganglia as tumor origin in the filum terminale or cauda equina have not been described.

Comprehensive molecular profiling of thoracic and abdominal PGL/PCC by the The Cancer Genome Atlas (TCGA) revealed a considerable diversity of driver alterations that resulted in the establishment of molecularly defined subtypes with prognostic impact [6]. Using DNA sequencing, gene expression profiling and methylation analysis, PGLs could be divided into four molecular groups: pseudohypoxic, cortical admixture, Wnt signaling, and kinase signaling [5, 6]. Approximately 40% of patients carry germline mutations in one of 19 PGL/PCC susceptibility genes (*DNMT3A, EGLN1, EGLN2, EPAS1, FH, IDH1, KIF1B, MAX, MDH2, NF1, SDHA, SDHAF2, SDHB, SDHD, SDHC, SLC25A11, RET, VHL, and TMEM127*), which are associated with a variable risk of metastatic disease [2, 5, 18].

The extent of methylation in PGLs ranges from genome-wide hypomethylation to hypermethylation [12]. Tumors were shown to belong to three methylation subclusters (M1–M3) which are strongly associated with molecular subtypes: pseudohypoxic PGLs with genetic alterations in tricarboxylic acid cycle genes show DNA hypermethylation (TCGA M1 cluster), pseudohypoxic VHL- and EPAS1-related PGL show intermediate DNA methylation (TCGA M2 cluster), whereas PGLs with Wnt and kinase signaling alterations exhibit DNA hypomethylation (TCGA M3 cluster) [6, 12].

Genetic analyses of patients with head and neck PGLs (HN-PGLs) have revealed similar driver mutations as in abdomino-thoracic PGL/PCC (mainly *SDHB, SDHD, VHL* mutations); however, methylation analysis was only conducted in very few cases [2, 24, 25, 27]. Genetic and epigenetic alterations in CEPs have not been comprehensively analyzed so far. Here, we analyzed a series of 57 cauda equina paragangliomas by genome-wide DNA methylation profiling and whole exome DNA and RNA sequencing. Comparing molecular signatures to those of non-spinal PGLs, including abdomino-thoracic and head and neck PGLs, we provide evidence that CEP represents a molecularly distinct tumor entity characterized by a unique epigenetic profile, unrelated to that of non-spinal PGLs, as well as the absence of common PGL-associated genetic driver alterations including germline mutations.

## Materials and methods

### Sample selection and histology

Tumor samples of 56 patients with the diagnosis of a CEP (*n* = 60; 55 primary tumors and 5 recurrent tumors) were collected from the archives of Neuropathology Departments of Hannover Medical School (MHH), Heidelberg University Hospital and Charité-Universitätsmedizin Berlin. We additionally included a cohort of head and neck paragangliomas (*n* = 24) which had not been previously analyzed within the TCGA framework. Histopathology was reviewed according to the current 2016 WHO classification of CNS tumors by at least two certified neuropathologists. Patients’ sex, age at diagnosis, tumor location, and follow-up data were obtained from local clinical records. Tissue and blood collection and processing as well as data collection complied with local ethical regulations, with extended informed consent given by study participants donating blood for sequencing. Ethical approval (EA2/113/18) was granted by the Charité Ethics Committee.

### Immunohistochemical procedures

Immunohistochemical stainings were performed on a Benchmark XT autostainer (Ventana Medical Systems, Tuscon, AZ, USA) with standard antigen retrieval methods (CC1 buffer, pH8.0, Ventana Medical Systems, Tuscon, AZ, USA) using 4-μm-thick, FFPE tissue sections. The following primary antibodies were used: monoclonal mouse anti-MIB1 (Ki67, 1:100, clone M7240, Dako), monoclonal mouse anti-cytokeratin (AE1/AE3, 1:200, clone M3515, Dako), monoclonal mouse anti-CK18 (1:1000, clone DC-10, BioGenex), monoclonal mouse anti-SDHB (1:100, clone 21A11AE7, abcam), monoclonal mouse antibody GATA3 (1:250, clone L50-823, Cell Marque).

### DNA extraction and DNA methylation analysis

Genomic DNA was extracted from formalin-fixed and paraffin-embedded (FFPE) samples and blood using the Maxwell 16 Blood and Tissue DNA Purification Kit (Promega) according to the manufacturer´s protocol. In addition to publically available methylation profiles of 20 CEPs (GSE109381), DNA methylation analysis was performed of 37 cauda equina paragangliomas (35 primary tumors, two recurrences) and 24 head and neck paragangliomas using the Illumina Infinium Methylation EPIC array as previously described [4].

### DNA and RNA sequencing

Whole exome sequencing of paired FFPE tumor DNA and blood leucocytes DNA was performed of a subset of ten cauda equina paragangliomas using the Agilent SureSelect human all exon V7 combined with the SureSelect^XT^ low-input reagent kits on a NovaSeq 6000 (Paired-End, 100 bp, S1) at the Genomics and Proteomics Core Facility of the German Cancer Research Center (DKFZ) in Heidelberg. RNA was extracted using the Maxwell 16 LEV RNA FFPE Kit (Promega) on a Maxwell 16 Instrument (Promega) following the manufacturer’s instructions. mRNA sequencing of FFPE-derived RNA was conducted according to a previously published FFPE RNA sequencing protocol on an Illumina NextSeq 500/550 instrument [34].

### Public datasets

Methylation data of pheochromocytomas (n = 143) and extra-adrenal paragangliomas (*n* = 29) were downloaded from the TCGA repository (https://portal.gdc.cancer.gov/projects/TCGA-PCPG). Publically available DNA methylation datasets were retrieved from the GEO Gene Expression Omnibus repository: paragangliomas (*n* = 18, GSE111336) [27], neuroblastoma (*n* = 105, GSE73515; *n* = 34, GSE120650), schwannoma (*n* = 125, GSE79009), melanoma (*n* = 37, GSE108576), desmoplastic melanoma (*n* = 15, GSE112308), superficial MPNST (*n* = 15, GSE112308), and pancreatic neuroendocrine tumors (*n* = 31, GSE117852). Additionally, the following tumor classes of the v11b4 reference set of the brain tumor classifier were included (GSE109381, [4]): cauda equina paraganglioma (*n* = 20), schwannoma (*n* = 23), melanoma (*n* = 12), melanocytoma (*n* = 15), hemangioblastoma (*n* = 25), myxopapillary ependymoma (*n* = 25), spinal ependymoma (*n* = 26), spinal subependymoma (*n* = 9), pituitary adenomas (*n* = 94). IDAT files of ganglioneuromas (*n* = 10) were provided by D. E. Reuss (previously published, [29]). Datasets were screened for low quality samples. Cases with a mean detection *p* value > 0.01 were excluded from further analyses.

### Bioinformatics and data analysis

#### Methylation analysis

DNA methylation data were analyzed using the R/Bioconductor package minfi (version 1.30.0) [1]. The intersection of CpG sites which are available on both methylation array types (450 k or EPIC) were combined applying the combineArrays function. The following filtering criteria were applied: removal of probes targeting the *X* and *Y* chromosomes, removal of probes containing a single-nucleotide polymorphism (dbSNP132 Common) within five base pairs of and including the targeted CpG site, and probes not mapping uniquely to the human reference genome (hg19) allowing for one mismatch. For *t*-Distributed Stochastic Neighbor Embedding (tSNE) analysis, the 32,000 most variable methylation probes across the whole dataset were analyzed using the Rtsne package (v0.15). The first 45 principal components (PCs) were calculated with singular value decomposition (SVD) and then analyzed by tSNE with the following parameter: pca = F, max_iter = 1000, theta = 0.5, perplexity = 45. For unsupervised hierarchical clustering analysis, we selected the 10,000 most variably methylated CpG sites across the dataset and used ComplexHeatmap (v2.3.1) with Euclidean distance and average linkage method in R. Copy number variations were calculated from IDAT files using the R/Bioconductor package conumee (https://bioconductor.org/packages/ release/bioc/html/conumee.html). DNA methylation-based classification was performed with the Heidelberg Brain Tumor Classifier (version v11b4) [4].

DNA whole exome sequencing data were processed via a customized bcbio-nextgen pipeline (v1.1.7-b) (https://github.com/bcbio/bcbio-nextgen). Reads were aligned to the hg19 human reference genome (GRCh37) using the Burrows–Wheeler Aligner (bwa, version 0.7.17). Duplicate reads were removed using biobambam (v2.0.87). Vardict (vardict-java, v1.6.0), mutect2 (part of gatk4, v4.1.4.0) and strelka2 (v2.9.10-0) were implemented as variant callers. Applying the ensemble option, only variants called by at least two variant callers were kept for further analysis. VCF file annotation was done using the variant effect predictor (vep; v100.1). ClinVar (v20200706) was included for custom annotations. Variants were classified into the three categories “likely benign/benign”, “uncertain significance” and “likely pathogenic / pathogenic” according to the joint AMP-ASCO-CAP 2017 guidelines for cancer variant interpretation with CancerVar (https://github.com/WGLab/CancerVar). We filtered for non-synonymous variants with an allele frequency of > 15% in coding regions with a maximum population allele frequency of less than 0.01%. Variants with the ClinVar annotation “benign” or “likely benign” or CancerVar verdict “likely benign / benign” were discarded. All cases were additionally analyzed via the Molecular Health platform (MH Guide, platform build 4.1.0, filter setting: same as above including coding regions, splice site, and extended splice site). PGL/PCC susceptibility genes (*DNMT3A, EGLN1, EGLN2, EPAS1, FH, IDH1, KIF1B, MAX, MDH2, NF1, SDHA, SDHAF2, SDHB, SDHD, SDHC, SLC25A11, RET, VHL, and TMEM127*) were manually inspected for exon deletions and exon–intron boundary alteration in each sample using the integrative genomics viewer (IGV, v2.8.6).

For RNA sequencing data analysis, a total of five different tools were used to identify fusions: Arriba (v1.1.0, https://github.com/suhrig/arriba/), EricScript (v0.5.5b) [3], FusionCatcher (v1.00, https://code.google.com/p/fusioncatcher/), InFusion (v0.8) [20], and STAR-Fusion (v1.8.0, https://star-fusion.github.io/). Arriba was run with default parameters except for –peOverlapNbasesMin 10 and –alignSplicedMateMapLminOverLmate 0.5 which was suggested to increase sensitivity [32]. STAR-Fusion was set to –peOverlapNbasesMin 10 with the remaining parameters in default. InFusion was used with the following non-default parameter: –min-fragments 3. Fusion candidates predicted by EricScript were discarded if the internal classification score (‘EricScore’) was ≤ 0.5 to better discriminate between true- and false-positive calls as recommended by the authors. The hg38 human reference genome was used for alignment. Fusion candidates were annotated using the FusionHub database [22]. Candidate fusions were filtered using the following set of criteria: Only fusions (i) predicted by ≥ 2 tools, (ii) with a read support > 10, and (iii) not annotated as read-through transcripts were kept. Gene fusions (iv) detected in non-neoplastic tissues curated in the FusionHub database (1000genome, Bodymap2, HPA, Non_Tumor_Cells, Babiceanu, Banned and GTEx), (v) with uncertain breakpoint prediction and detected in control tissue were discarded (Supplementary Figure S1, online resource). The final fusion list was annotated with oncofuse and filtered according to a high driver probability score (> 0.9) and a low passenger score (< 0.5) [30].

### Statistical analysis

For patients with available follow-up information, overall survival (OS) was calculated from the time of the initial histological diagnosis to last contact or death. Recurrence was defined as the time-point of radiological tumor reappearance in CEPs and the occurrence of an aggressive disease event (local recurrence or metastasis) in non-CEPs from the TCGA dataset. Statistical and survival analyses were performed in R applying the packages “survminer” (v0.4.6) and “ggpubr “ (v0.2.4). Differences in progression-free survival (PFS) and OS were tested for statistical significance using the log-rank test. The significance level was set at *p* < 0.05.

## Results

### CEPs are benign, sporadic tumors that rarely recur or metastasize to the CNS

Analysis of clinical data of 56 CEP patients demonstrated that 37 (66%) CEPs occurred in males and 19 (34%) in females, with a male predominance M/F = 1.95/1, in line with previous reports [16, 33]. The age of patients ranged from 18 to 78 years (Supplementary Table S1, online resource). All primary lesions occurred between L1 and S2 with five tumors arising from the filum terminale and 12 from cauda equina nerve roots. CEPs usually presented as solitary, contrast-enhancing masses (Fig. 1c, d).

In our series, five CEPs recurred (9%) and two tumors recurred twice over a period of 10 years (CEP11) and 20 years (CEP2), respectively. In four patients (7%), a disseminated manifestation was observed (two cases at initial diagnosis, two cases upon recurrence). CEP35 recurred 6 years later with multiple lesions in the cauda equina and thoracic spine and developed a cerebellar metastasis 18 years after initial diagnosis (Fig. 1e–f). No distant metastasis outside the CNS was observed in any of the CEP cases. None of the CEP patients had a family history of PGL or presented with an additional primary PGL outside the CNS (*n* = 39, NA = 17).

Follow-up data were available for 37 patients. The median follow-up time was 65 months (range: 2 months–31 years). Overall, two patients died of unknown reasons 31 years and 20 years after initial presentation, respectively. We compared PFS and OS between CEPs, extra-adrenal PGLs and pheochromocytomas, but did not observe a significantly different outcome in patients with CEPs and PGLs (Supplementary Figure S2, online resource).

### Cauda equina paraganglioma is a distinct molecular tumor entity defined by a unique methylation profile

**Fig. 2:**
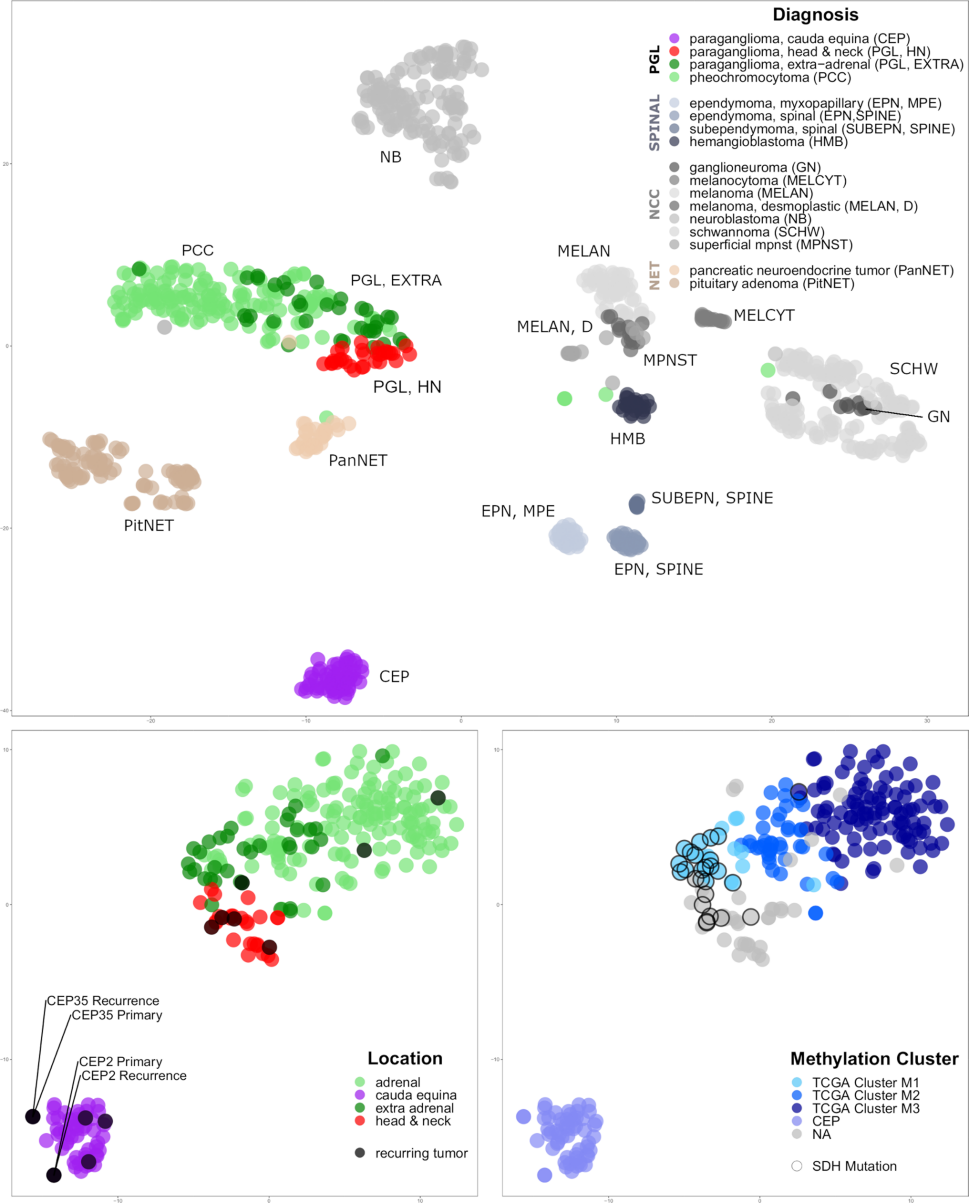
Cauda equina paragangliomas have distinct epigenetic profiles. **a** DNA methylation-based tSNE analyses of paragangliomas and neural crest cell-derived tumor entities as well as relevant differential diagnoses of the spinal compartment: cauda equina paragangliomas form a distinct group separate from all other tumor entities. **b** While pheochromocytomas, extra-adrenal and head and neck paragangliomas group together, no overlap with cauda equina paragangliomas can be observed. Recurring paragangliomas (including the cerebellar metastasis in CEP35) are not different from primary paragangliomas and merge with their epigenetic subgroup. **c** Head and neck PGLs are located close to the TCGA M1 hypermethylated group enriched for tumors with SDH mutations (circled). *PGL* paraganglioma subtypes, *SPINAL* relevant differential diagnosis in spinal location, *NCC* neural crest cell-derived tumor entities, *NET* neuroendocrine tumors

We compared genome-wide methylation profiles of 57 CEPs (55 primary tumors and 2 recurrent tumors) from 56 patients with previously published abdomino-thoracic PGL/PCC (*n* = 185) [6]. We additionally profiled 24 HN-PGLs including 15 glomus jugulare tumors, 2 glomus jugulotympanic tumors, 1 glomus tympanicum tumor, 6 glomus aorticum tumors, and 1 tumor of the glossopharyngeal nerve. We also included 14 neural crest cell-derived tumor entities as well as relevant differential diagnoses of the lower lumbar spine. On *t*-distributed Stochastic Neighbor Embedding (tSNE) analysis, CEPs clearly grouped apart from PGLs of other anatomical locations showing distinct epigenetic profiles (Fig. 2a). In contrast, methylation profiles of HN-PGLs intermingled with abdomino-thoracic PGL/PCC but showed no overlap with CEPs.

**Fig. 3:**
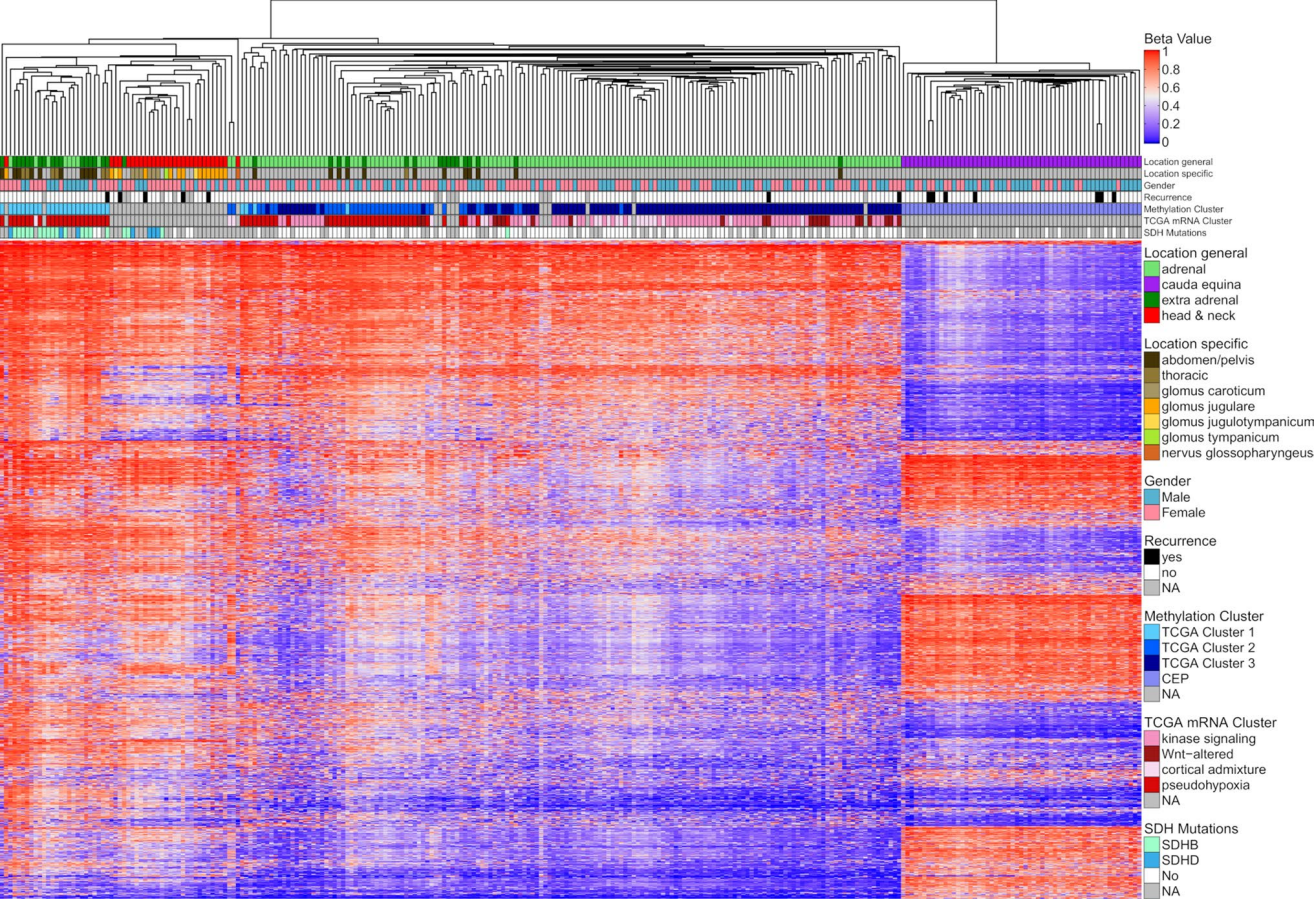
Heatmap of paragangliomas of various locations. Unsupervised hierarchical clustering based on the 10.000 most differentially methylated CpG sites clearly separates CEP from other paragangliomas. Furthermore, TCGA subclusters within paragangliomas of thoraco-abdominal and adrenal origin is recapitulated (M1-M3 cluster). NH-PGLs form a separate subcluster close to M1 hypermethylated cases within PGLs

Recurrent tumor samples grouped in proximity with their primary tumors on tSNE analysis, including a metastasis of CEP35 in the cerebellopontine angle, which was initially suspected to be a glomus tumor (Figs. 1f, 2b). Furthermore, tSNE analysis of methylation profiles of CEPs and PGLs only revealed that HN-PGL integrated in the extra/adrenal PGL group and aggregated in proximity to hypermethylated TCGA PGLs (M1 Cluster), enriched for tumors of the molecular subgroup pseudohypoxia with frequent SDH mutations (Fig. 2c). The epigenetic difference between CEPs and PGLs could also be shown by unsupervised hierarchical clustering in a heatmap displaying the 10.000 most differentially methylated CpG sites (Fig. 3). CEPs formed a distinct cluster while HN-PGLs clustered close to PGLs.

### Cauda equina paragangliomas are characterized by balanced copy number profiles and rarely show loss of chromosome 3

**Fig. 4:**
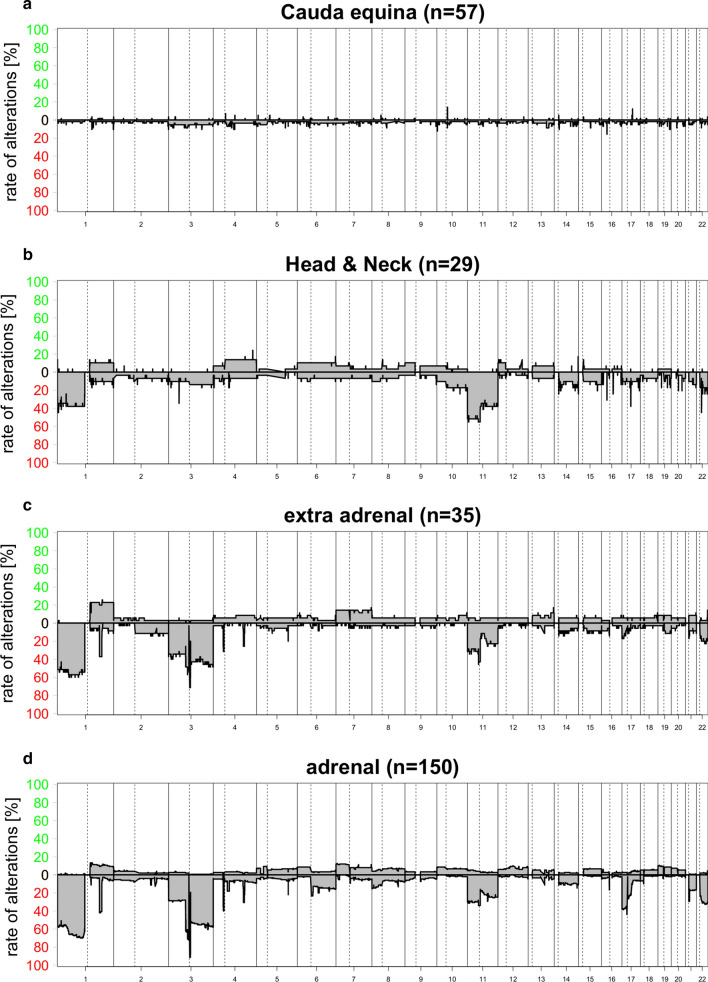
Chromosomal copy number (CNV) summary plots for the PGL subtypes: CEPs (**a**), HN-PGLs (**b**), extra-adrenal PGLs (**c**), PCC (**d**). Chromosomal alterations are largely missing in CEPs compared to other PGLs. Extra-adrenal PGLs and PCC show similar frequencies of losses of chromosomes 1p, 3 and 11 whereas HN-PGLs rarely show chromosome 3 loss

We analyzed copy number variation (CNV) profiles calculated from DNA methylation array data of 57 CEPs (Fig. 4a). CNV plots did not show chromosomal alterations—including amplifications or deletions—except for CEP18 and CEP55, both demonstrating a loss of chromosome 3. In contrast, copy number profiles of HN-PGLs, extra-adrenal PGLs, and PCCs showed frequent chromosomal aberrations (Fig. 4b–d), mostly affecting similar chromosomes. Chromosome 1p (including *SDHB*) loss was observed in 40–60% of PGLs and chromosome 11 (including *SDHD*) loss occurred in 30–40% of PGLs. Extra-adrenal PGLs and PCCs were enriched for chromosome 3 (including *VHL*) losses (40–50%) as compared to HN-PGLs (10–15%). Loss of chromosome 17p (including *TP53*) was more often observed in PCCs (40%) compared to extra-adrenal PGLs and HN-PGLs (10%).

### Mutations in PGL/PCC susceptibility genes are absent in CEPs

We performed whole exome sequencing of 10 CEPs and matched normal controls. On average, 77 million reads (range 63–93 million reads per sample) were generated, resulting in a median real coverage of 94X per sample (range 77–122 per sample). After filtering variants detected by our in-house pipeline, a total of 33 non-synonymous single-nucleotide variants (SNVs) and insertion/deletions (indels) in coding regions and splice sites were identified (3.3 ± 2.5 (mean ± SD) alterations per tumor). None of the variants were classified as likely pathogenic or pathogenic according to the joint AMP-ASCO-CAP 2017 guidelines for cancer variant interpretation. We additionally analyzed all cases with the same filter setting via MH Guide: eight variants of uncertain significance remained. Variants classified as “uncertain significance” were reviewed in varsome (https://varsome.com) [10], none was reported to occur in the context of cancer (Supplementary Table S2, online resource). Additionally, we manually reviewed all PGL/PCC susceptibility genes for exon deletions and intron–exon boundary alterations, including synonymous SNVs at splice sites, but did not find a pathogenic alteration in any of the cases.

### CEPs are not driven by recurrent oncogenic gene fusions

Fusion genes involving *MAML3* (5%), *BRAF* (0.6%), *NGFR* (0.6%), and *NF1* (0.6%) have been described as genetic disease drivers in a subset of paragangliomas [6]. We, therefore, performed RNA sequencing and analyzed eight CEPs. Application of the filter setting resulted in one final candidate fusion (Supplementary Figure S1, online resource). The fusion did not involve genes being described as cancer relevant drivers and was not observed in other CEPs. Additionally, the raw output of all fusion callers was screened for fusions containing *MAML3*, *BRAF*, *NGFR*, or *NF1*. The fusion partners of these genes identified this way have not been described before and their predicted break points deviated from the ones identified in literature [6, 38]. A low read support and the fact that they were called by a single fusion caller only, suggests that these fusions are artificial. In summary, fusion genes known to be relevant in PGL/PCC and associated with poor prognosis (e.g. *MAML3* fusions) were not observed in CEPs.

### CEPs show a wide spectrum of morphological appearances with a high intratumoral heterogeneity

**Fig. 5:**
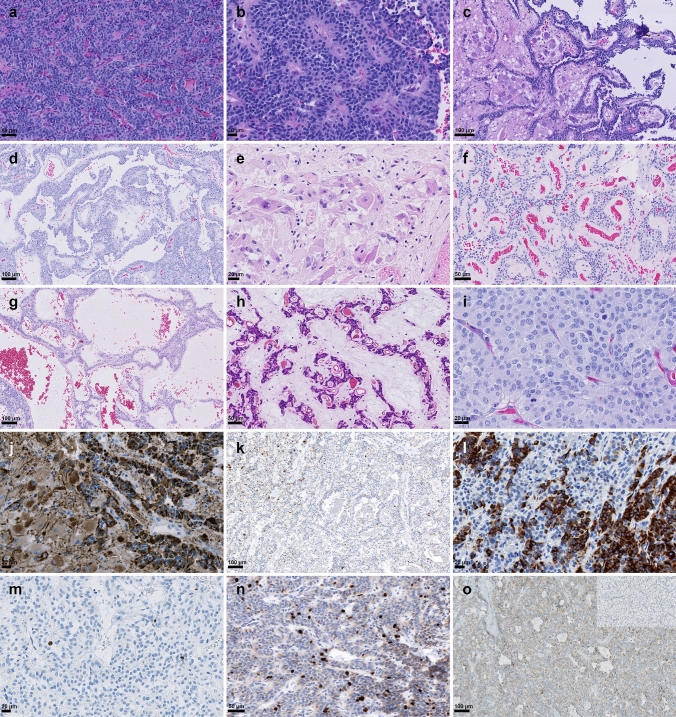
Histopathology of cauda equina paragangliomas. Hematoxylin and eosin (H&E) stain demonstrates a monomorphic tumor with neuroendocrine differentiation and “zellballen” growth pattern (**a**). The same tumor (CEP30) demonstrated alternating areas with perivascular pseudorosettes (**b**), papillary formations (**c**) and a ganglioneuromatous component (**d**). CEPs can also present with predominantly gangliocytic differentiation (**e**) or angiomatous differentiation (**f**), sinusoidal appearance (**g**) and strongly hyalinized fibrovascular stroma (**h**). Focally increased mitotic activity with several mitoses was observed in some cases (**i**). Strong, granular, and dot-like pan-cytokeratin expression in epithelioid and gangliocytic cells (**j**), sometimes present only in single cells that may easily be missed (**k**). Patchy cytokeratin CK18 expression in a CEP (**l**). Proliferation activity indicated by ki67 immunohistochemistry (IHC) is usually low (**m**), but may be increased in a heterogenous pattern throughout the tumor (**n**). Cytoplasmic SDHB staining was retained in tumor cells in all cauda equina paragangliomas (**o**), loss of SDHB expression is shown for a HN-PGL in the inset

PGLs usually show a classic organoid “zellballen” growth pattern that was at least focally present in all CEPs (Fig. 5a). In addition, most CEPs (*n* = 54) demonstrated a wide spectrum of growth patterns and presented with high intratumoral heterogeneity with several different alternating growth patterns observed within one tumor (e.g. CEP30, Fig. 5a–d). We observed perivascular pseudorosettes (*n* = 37, Fig. 5b), papillary formations (*n* = 15, Fig. 5c), ganglioneuromatous differentiation (*n* = 4, Fig. 5d), gangliocytic maturation (*n* = 11, Fig. 5e) and angiomatous or sinusoidal growth pattern occasionally with strong hyalinization (n = 16, Fig. 5f–h) in CEPs (Supplementary Table 1, online resource).

### CEPs are characterized by a heterogenous mitotic and proliferative activity

Mitotic count per 10 high power fields (HPF) ranged from 0 up to 5 in CEPs. In some cases, several mitoses per HPF were observed (Fig. 5i). Overall, the mitotic activity was highly variable throughout the tumor tissue, which was also reflected by a very heterogeneous proliferation activity (Ki67 immunohistochemistry). Most CEPs had a low proliferation activity with Ki67 being 0–3% in most tumor areas (Fig. 5m); however, 25 tumors repeatedly showed areas with focally increased proliferation (Ki67 5–10%, Fig. 5n). Most tumors that recurred or demonstrated spinal metastasis (4 of 5), did not demonstrate an increased proliferation or mitotic count. Moreover, we did also not observe an association of a distinct growth pattern with increased proliferation or mitoses.

### Cytokeratin and SDHB is expressed in all CEPs, whereas GATA3 expression is absent

Compared to HN-PGLs which were all negative for cytokeratin expression (*n* = 24), we detected pan-cytokeratin (AE1/AE3) immunoreactivity in all CEPs (*n* = 59, NA = 1). Cytoplasmic-granular and/or dot-like staining pattern was homogenously observed in all tumor cells in 37 CEPs (63%, Fig. 5j), being expressed by the epitheloid as well as the gangliocytic cells. A patchy staining pattern with clusters of positive cells was observed in 13 CEPs (22%). In 9 cases (15%), we detected only single positive cells diffusely dispersed within the tumor (Fig. 5k). To further investigate if a specific subtype of keratin is preferentially expressed, we stained different cytokeratins (e.g. CK5/6, CK7, CK18, CK19, and CK20). The only cytokeratin being expressed in the same staining pattern as AE1/AE3 was CK18 (positive in 7/7 CEPs, negative in 7/7 HN-PGLs, Fig. 5l). A patchy cytokeratin expression was not observed in cases that demonstrated spinal dissemination, but in two cases that recurred (CEP11, CEP35). We additionally analyzed CEPs for *SDHB* mutations by immunohistochemistry. None of the CEPs demonstrated a loss of cytoplasmic SDHB expression (*n* = 35, NA = 25; Fig. 5o). GATA3 nuclear expression was seen in the majority of HN-PGLs (17/21, NA = 4), but absent in all CEPs analyzed (*n* = 31, NA = 29).

## Discussion

In this study, we analyzed a large cohort of CEPs using genome-wide DNA methylation analysis as well as whole exome DNA and RNA sequencing. Comparing our results to paragangliomas of abdominal, thoracic and head and neck origin, our data suggest that CEP is an independent tumor entity with distinct molecular, histopathological, and clinical features.

Methylome analysis has been of great interest in PGLs even before the rise of methylation-based classification in CNS tumors [4, 6, 12]. Besides revealing a cell-of-origin signature reflected in the methylation pattern of a tumor, epigenetic profiling also proved powerful in predicting the occurrence of genetic drivers in some tumor entities (e.g. CpG island methylator phenotype (CIMP) in IDH mutant gliomas) [19]. In PGLs, methylation analysis identified three stable clusters associated with clinical features and mutational status. For example, the hypermethylation phenotype (Cluster M1) is observed in tumors with TCA cycle-related genes (hereditary in 100%) and associated with a high risk of metastatic disease whereas tumors with Wnt signaling alterations (CSDE1, MAML3) belong to the hypomethylated M3 cluster (hereditary in 0%) [5, 6].

As a reference cohort, few CEPs have been epigenetically profiled within the Heidelberg brain tumor classifier project [4]. According to the current opinion, they are most likely related to the hypomethylated M3 cluster (methylation class "paraganglioma, spinal non-CIMP", https://www.molecularneuropathology.org/mnp/classifier/2/group/289). However, analyzing a large cohort of CEPs by methylation profiling and comparing them to PGLs of other sites, we noted that CEPs were epigenetically very distinct from other PGLs and formed an independent methylation cluster. Distinctiveness of methylation pattern in CEPs vs. PGLs were also demonstrated by high calibrated classifier scores (> 0.93) for CEPs (*n* = 38) whereas none of the HN-PGL (*n* = 25), PCC (*n* = 4) and extra-adrenal PGL (*n* = 4) were classifiable by the DKFZ CNS tumor classifier v11b4 (all calibrated classifier scores < 0.7).

HN-PGLs share the same mutational and epigenetic alterations as PGL/PCC and clearly belong to the large group of PGLs of abdomino-thoracic origin. The high influence of similar driver genes on methylation patterns independent of location was also shown for atypical teratoid rhabdoid tumors (ATRTs) and non-central nervous system malignant rhabdoid tumors (extra-CNS MRTs) that are defined by SMARCB1 alterations and were mostly classified as AT/RT, MYC subgroup (85%) [26]. In contrast, CEPs do not cluster with PGLs of other sites, suggesting another molecular background in CEPs.

Indeed, we did not find any of the recurrently altered driver genes in PGLs by DNA and RNA exome sequencing and demonstrate absence of SDHB mutations in CEPs by immunohistochemistry. We especially did not find germline mutations present in up to 40% of PGLs, further providing evidence for a sporadic development of CEPs. These molecular findings are in line with our clinical follow-up data: none of our CEP cases had a family history of PGL syndrome, a second primary PGL or occurrence of an increased number of other tumors (one patient had breast cancer and one patient had colon carcinoma) and all tumors occurred in adults.

A primarily sporadic origin of CEP has been suggested by several authors before [13]. In contrast, Masuoka et al. described in 2001 the first CEP with a missense SDHD mutation in codon 12 (GGT–AGT, Gly–Ser) [15]. The tumor recurred 22 years later as a cerebellar metastasis and both tumors were found to carry the same SDHD mutation suggesting an inherited origin. According to recent databases, the reported SDHD mutation (SDHD c.34G > A;p.Gly12Ser, NM_003002.4; rs34677591) is currently believed to be a benign SNP (GnomAD exomes allele frequency = 0.00749).The authors additionally screened 22 CEPs for SDHD mutations but did not identify any further mutation. In 2008, Persu et al. [24] sequenced coding regions of *SDHB, SDHD, RET*, and *VHL* in two sporadic CEPs and pathogenic mutations were found to be absent. Except for one CEP that was subjected to comparative genomic hybridization (CGH) and showed no copy number changes [9], we did not find further published cases with genetic information. Although, available evidence is limited, a CEP with hereditary origin has not been described to date which is supported by our data.

Based on unsupervised clustering of methylation profiles, we did not find evidence for subclusters within CEPs as was seen for PGLs. We, therefore, assume that the molecular background among CEPs is very similar and does not reflect the mutational diversity observed in PGLs [6]. Based on DNA and RNA sequencing, we did not find a recurrent driver mutation or fusion in CEPs. Similarly, in central neurocytomas, sharing an unknown cell-of-origin, neuronal phenotype and rarely gangliocytic differentiation with CEPs genetic drivers are currently unknown [13]. Further analysis, including regulatory non-coding RNAs and post-transcriptional modifications, will likely be necessary to identify the driver alteration of CEPs as well as the analysis of primary epimutations and strand-specific methylation patterns, as imprinting seems to play a pivotal role in disease penetrance in paraganglioma cells in a syndromic context [18].

CEPs are usually benign with local recurrence rates of 4% [13, 17, 37] and few cases showing cerebrospinal fluid dissemination and seeding to the CNS [28, 35, 36]. However, in our large series, we noted a recurrence rate of approximately 9% (5 of 56 patients) with spinal dissemination in four of our cases possibly indicating an underestimated risk of recurrence. CEP35 presented with a metastasis in the cerebellopontine angle 18 years after initial diagnosis. Methylation and copy number profiles did not change over almost two decades and allowed to clearly differentiate between a cerebellar metastasis of CEP vs. a new primary tumor. Histological features associated with a more aggressive clinical course have not been identified in CEPs [13] which is in line with our results showing that increased mitotic or proliferative activity was not associated with a higher risk of recurrence or dissemination. In PGLs, molecular stratification proved superior in predicting clinical outcome [5, 6, 18], which is not possible in CEPs based on DNA methylation analysis so far and needs to be further investigated.

In our series, CEPs presented with a remarkable diversity of morphological growth patterns with high intratumoral heterogeneity. Some tumors even showed capability of gangliocytic and ganglioneuromatous differentiation, suggesting a high potential for transformation or maturation rarely observed in PGLs of other sites. The difference between CEPs and PGLs was further underlined by GATA3 expression pattern. Nuclear GATA3 expression has been shown in 75% of extra-adrenal PGLs and 71% of PCCs [23, 31]. In contrast, GATA3 was not expressed in any of the 31 CEPs analyzed of our own cohort. In line with our own findings, Mamilla et al. [14] recently reported absence of GATA3 expression in three CEPs as well as strong cytokeratin expression. Compared to previous studies showing cytokeratin expression in some CEPs [11, 17, 21], all CEPs in our series strongly expressed cytokeratin. However, in some cases, expression was restricted to some tumor areas or limited to a few cells within the tumor prone to be missed. The multiple morphological facets of CEPs together with its unusual cytokeratin expression pattern and rarity bear a risk for misdiagnosis or assumption of collision tumors. Methylation profiling proved capable of differentiating CEPs from other cytokeratin expressing neuroendocrine tumor entities (e.g. pancreatic neuroendocrine tumors, pituitary adenomas) and morphological mimics of the lower spinal cord (e.g. ependymomas, spinal metastasis).

The histogenesis of CEPs is not well established. It is currently unclear if precursor paraganglia cells are a normal component of the cauda equina or whether a disrupted migration of neuroblasts during development resulted in displaced precursor cells in an unintended microenvironment [13, 16, 28]. A common ancestral cell of all PGLs is very likely, given their similar morphology and ultrastructure irrespective of sites of origin [18]. Methylation profiling—known to particularly reflect a cell-of-origin signature—suggests the same origin of paragangliomas occurring in the head and neck and the abdominal region. These PGLs develop from different subpopulations of migrating neural crest cells (e.g. cranial, vagal and trunk) and belong to the same methylation group, despite multiple genes and pathways involved in their formation.

In contrast, CEPs are epigenetically very distinct which might not be completely explained by the development of a sacral neural crest cell subpopulation and another molecular background. Moreover, CEPs in contrast to PGLs strongly express cytokeratin. Expression profiling of newly induced neural crest cells suggests a model in which migratory potential is acquired in pre-migratory neural crest cells by a sequential change in the type of cytoskeletal elements expressed [8]. Intermediate filament genes (e.g. CK18) are characteristic for early pre-migratory neural crest cells, whereas they are lost in migrating neural crest cells expressing actin cytoskeleton elements instead. Morphological similarity and epigenetic differences in CEPs and PGLs might possibly be attributed to formation from neural crest cells precursor cells in different developmental states (early pre-migratory vs. migratory neural crest cells).

## Electronic supplementary material

Below is the link to the electronic supplementary material.

Supplementary Table 1 Summary of clinical, morphological and immunohistochemical characteristics in CEPs

Supplementary Table 2 Summary of filtered DNA sequencing results containing variants of uncertain significance

Supplementary Figure S1 Flowchart of the RNA fusion calling pipeline

Supplementary Figure S2 Kaplan-Meier survival curves of patients with CEP, extra-adrenal paragangliomas and pheochromocytomas. Progression-free (a) and overall (b) survival was not significantly different between CEPs, pheochromocytomas and extra-adrenal paragangliomas
