# Supplementary figures and images for: Molecular characterization of CNS paragangliomas identifies cauda equina paragangliomas as a distinct tumor entity
Source: Acta Neuropathol. 2020 Sep 14;140(6):893–906. doi: 10.1007/s00401-020-02218-7 (PMC7666289; doi:10.1007/s00401-020-02218-7)

Supplementary Figure 1

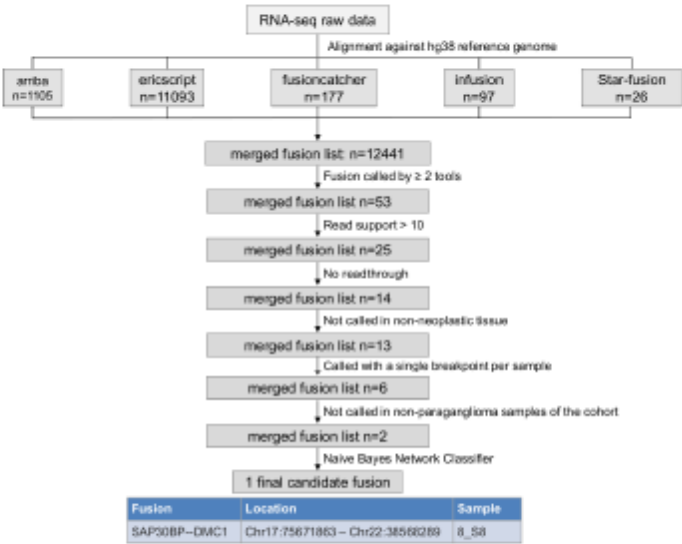

Supplement: Supplementary file 3 — Supplementary Figure S1 Flowchart of the RNA fusion calling pipeline [file 401_2020_2218_MOESM3_ESM.pdf]

Supplementary Figure 2

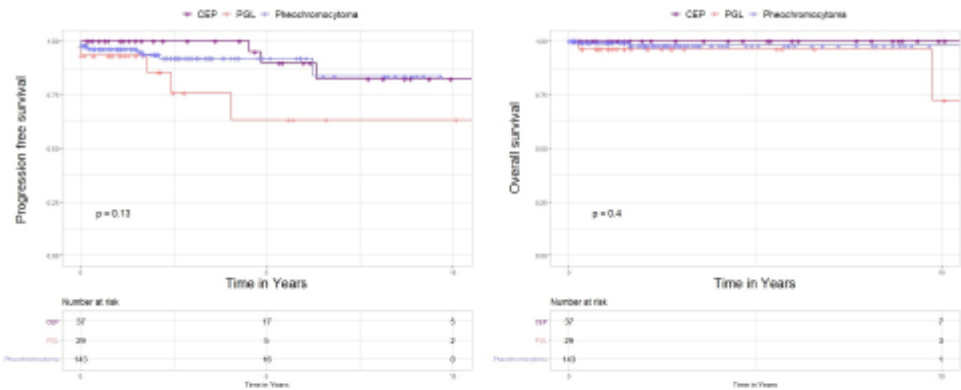

Supplement: Supplementary file 4 — Supplementary Figure S2 Kaplan-Meier survival curves of patients with CEP, extra-adrenal paragangliomas and pheochromocytomas. Progression-free (a) and overall (b) survival was not significantly different between CEPs, pheochromocytomas and extra-adrenal paragangliomas [file 401_2020_2218_MOESM4_ESM.pdf]
